# Review of Mental Health Response to COVID-19, China

**DOI:** 10.3201/eid2610.201113

**Published:** 2020-10

**Authors:** Adriana Miu, Hui Cao, Bohan Zhang, Huaiyu Zhang

**Affiliations:** University of Texas Southwestern Medical Center, Dallas, Texas, USA (A. Miu);; Beijing Institute of Education, Beijing, China (H. Cao);; Lesley University, Cambridge, Massachusetts, USA (B. Zhang);; University of California San Francisco, San Francisco, California, USA (H. Zhang)

**Keywords:** mental health response, mental health services, coronavirus, viruses, coronavirus disease, COVID-19, severe acute respiratory syndrome coronavirus 2, SARS-CoV-2, respiratory infections, psychosocial support, zoonoses, China

## Abstract

Public mental health response to coronavirus disease is essential. After reviewing systemic and local efforts in China, we found efficient coordination and human resources. We recommend better symptom assessment, monitoring of organizations, and basic needs protection. This recommendation can inform how other countries can overcome mental health challenges during this pandemic.

The coronavirus disease (COVID-19) outbreak and quarantines have caused major distress in China (*1*,*2*). Therefore, effective public mental health response to COVID-19 is needed (*3*). We review systemic and local mental health efforts in China based on psychiatric emergency guidelines from the Inter-Agency Standing Committee (*4*). These guidelines are coordination between multiple sectors; human resources; assessment, monitoring, and evaluation; and protection and human rights standards. Our discussion will inform mental health response for the COVID-19 pandemic.

Mental health efforts in China have been coordinated and facilitated through multiple systems, including government, academic societies, universities, hospitals, and nonprofit organizations (*5*). Services include a countrywide 24/7 hotline, text support through apps, psychoeducation materials, and webinars (*5*). The government prioritized psychosocial support for COVID-19, as shown by the National Health Commission mandate requiring all mental health associations to provide psychosocial support, establish professional focus groups, and aid the provincial and city health departments (*6*).

Academic organizations in psychology (Chinese Psychological Society [CPS]) (Table) and psychiatry (Chinese Society of Psychiatry) provide evidence-based guidelines on psychosocial support and training (*5*,*7*). The Ministry of Education (MoE) has mandated all college counselors across the nation to volunteer for the primary Huazhong University hotline at the epicenter in Wuhan (*8*). At the systemic level, there is good coordination and resource allocation. The government agencies coordinate human resources, and academic associations provide professional knowledge and guidelines for frontline effort.

**Table:** Case-patients with coronavirus disease in China and respective response from the Chinese Psychological Society

Date	Coronavirus disease	Chinese Psychological Society response
2019 Dec 9	First suspected case	None
2019 Dec 31	Cluster of pneumonia cases in Wuhan	None
2020 Jan 20	Cases in China, Thailand, Japan, and South Korea	None
2020 Jan 21	Cases reported in other provinces	None
2020 Jan 23	Lockdown of Wuhan	None
2020 Jan 25	Cases in all of China except Tibet	None
2020 Jan 26	No event	Published self-help article on emotional support
2020 Jan 27	Lockdown of all cities in Hubei	None
2020 Jan 28	No event	Conducted first round of training for supervisors
2020 Jan 29	No event	Published list of psychologist consultants
2020 Jan 30	World Health Organization declared public health emergency of international concern	None
2020 Jan 31	No event	Published handbook for hotline organizations and volunteers
2020 Feb 2	No event	Published list of organizations for hotline and counseling
2020 Feb 3	No event	Updated guidelines for hotline organization
2020 Feb 5	Foreign airlines cancelled flights to China	None
2020 Feb 6	No event	Published ethics guidelines
2020 Feb 7	Death of whistleblower doctor	None
2020 Feb 7	No event	Published webinars for general public
2020 Feb 9	No event	Published handbook on self-care for volunteers
2020 Feb 10	No event	Conducted second round of training for supervisors
2020 Feb 13	No event	Published hotline support questions and answers
2020 Mar 3	No event	Updated handbook for hotline organizations and volunteers
2020 Mar 3	No event	Published list of 52 recommended hotline organizations
2020 Mar 8	No event	Conducted 7-day self-help psychosocial support for healthcare workers

Coordination and resource allocation were compiled from local efforts at the Wuhan epicenter (Appendix). On January 23, 2020, immediately after the quarantine, Zhongnan Hospital and the Hubei Psychological Consultant Association began offering hotline services. As of April 30, more than 2,000 persons had been served. Beyond the hotline, Wuhan University and Huazhong University provide online text support through apps staffed by >3,000 professionals across China. This support demonstrates how hospitals, professional associations, and universities have collectively provided immediate resources. Furthermore, resources have been mobilized from other regions to support the epicenter. The hotline of Huazhong University became the primary hotline for Hubei residents and was staffed by college counselors throughout China under the mandate of the MoE (*8*). Psychologists and nurses from other provinces were dispatched to Wuhan Third Hospital on January 28. Psychosocial efforts might be sourced by different organizations, but they illustrate pooling of resources and coordination from other regions to ensure access to psychosocial support at the epicenter.

The MoE and CPS recruited professionals and volunteers across China, which suggests adequate resource allocation (*5*,*7*,*8*). CPS trained 1,448 registered psychologists in train-the-trainer workshops (*8*); these psychologists in turn supervised and provided live consultations to frontline volunteers (*7*). China has also implemented Artificial Intelligence Tree Holes Rescue to reduce suicidal risk. These programs demonstrate efficient task-sharing, by pooling professionals together, supervising less-trained staff, and using technology to overcome resource shortages.

The Inter-Agency Standing Committee calls for assessment of mental well-being and program evaluation of psychsocial support effectiveness (*4*). Guidelines of the National Health Commission document the need for assessment and program evaluation, but enforcement was unclear beyond the guidelines (*6*). Although there were nationwide surveys of psychological well-being (*9*,*10*), they did not describe use of surveys in psychological services. Clinical assessment, such as previous mental illness history or stressors (e.g., grief, financial stress), should be routinely integrated into services.

CPS published a list of approved hotline organizations based on survey evaluation of organizations (*8*). However, this survey was not conducted until 3 weeks after the outbreak. At the outset of a psychiatric emergency, a team of professionals should evaluate and monitor whether individual organizations meet national guidelines. A negative experience from an unregulated organization can deter persons from seeking help.

Although COVID-19 does not cause intentional harm, there are human rights issues on access to basic needs (*4*). During the sudden lockdown of Wuhan, access to food and medical needs was threatened because of food hoarding, price gouging, and transportation freeze. In response, the government coordinated supply with tons of vegetables and meat. These threats were documented by nationwide surveys of well-being of persons. Professionals can further use these documentations to advocate for victims. For example, professionals can educate policymakers about the need for transparency, such as informing the public about food shortage while reassuring the public that supply will arrive in a few days. China has provided free, country-wide psychosocial support, funded by the government and institutions (*5*–*7*). The accessibility is remarkable compared with that in other countries that depend on health insurance benefits.

Our review suggests that China has overcome resource shortages with coordination and resource allocation in its mental health response. The government, universities, and academic societies provide coordination, and independent organizations provide local support. We recommend integration of assessment in direct support, monitoring of organizations, and advocating for affected persons. These recommendations can inform how other countries can overcome shortage of mental health resources when facing this pandemic.

Appendix Additional information on review of mental health response to COVID-19, China.
