# Supplementary material for: Review of Mental Health Response to COVID-19, China
Source: Emerg Infect Dis. 2020 Oct;26(10):2482–4. doi: 10.3201/eid2610.201113 (PMC7510735; doi:10.3201/eid2610.201113)
Supplement: Appendix — Additional information on review of mental health response to COVID-19, China. [file 20-1113-Techapp-s1.pdf]

Article DOI: https://doi.org/10.3201/eid2610.201113

Review of Mental Health Response to COVID-19, China

Appendix

Personal Perspectives

The sudden lockdown in Wuhan led to psychological distress (*1*–*3*). In response, there has been coordination of national and local mental health efforts across China and at the epicenter (Wuhan) (*4*–*12*). With the plethora of mental health resources described, little has been written about the firsthand experience and unique challenges from psychosocial support organizations. Below are perspectives from a frontline volunteer and the cofounder of Yong Xin Kang Yi (用心抗疫 [Fighting Coronavirus with Heart]), a major psychosocial support organization with >120 volunteers who are licensed professionals in China and overseas (*13*).

The cofounder was one of the coauthors (H.C.). Yong Xin Kang Yi is a volunteer-based organization that serves medical staff. We began recruitment on January 24 and the hotline started on January 27. With additional support from the community, we eventually added the following services: confidential texting, support for online medical group, webinars, online groups, self-care manuals, and psychoeducation for the public. Together, our organization has >200 volunteers, 20 clinical supervisors, and 100 administrative staff. Up until now, we have served 300 frontline healthcare staff and >10,000 persons viewed our webinars. We intervened in 3 suicidal crisis cases. During each hotline shift, there is a supervisor on-call to provide immediate support to the hotline volunteer.

We have encountered some challenges. The primary challenges were related to volunteers in terms of their limited time availability and limited experience in crisis intervention. Examples of other challenges include not having a toll-free hotline and not being listed on the national hotline registry until early March.

On the basis of experiences during the past 2 months, I have learned many lessons. It is useful to be agile and adaptable to the situations. For example, because medical staff were too tired to talk after their shift, we began providing support by texts. As a result, more persons used

our services. We immediately strengthened the crisis intervention skills of our volunteers with more training and supervision after someone showed a risk for suicide.

Because we also noticed grief and anger of volunteers in the wake of Dr. Li Wenliang's death, we facilitated group compassion exercises for the volunteers. These exercises were effective in reducing burnout. We found it helpful to guide our organization on the basis of the ethical value of Do-No-Harm and evidence-based knowledge. Despite various practical challenges, our organization worked to help our service users feel validated and supported.

Psychosocial support for a disease outbreak differs from that for other emergencies, such as a natural disaster or a terrorist attack. Because the coronavirus disease outbreak is an ongoing pandemic, rather than a discontinued event, it has complicated the ability to provide direct support to frontline staff. Given that this crisis has mixed natural and human causes, early psychological reactions include both anxiety toward outbreak uncertainties and also societal resentment. There are often new discoveries about this unknown disease, and this requires volunteers to constantly keep themselves up-to-date with new developments. Psychosocial support needs to be timely. Otherwise, it could contribute to societal panic and chaos. Volunteers and supervisors need to practice good self-care to prevent compassion fatigue. Psychosocial support during an epidemic requires quick decision-making and flexibility.

One of the coauthors (B.Z.) was a hotline volunteer from January through February. I received mostly text messages from ≈15 persons, and most of them were frontline medical staff and their family and friends. Presenting concerns included stress, anxiety, panic attacks, fear, anger, relationship conflicts, and helplessness. I practiced several effective approaches, such as empathic listening, normalization of feelings, and psychoeducation about stress reactions. Other approaches included motivational interviewing, and teaching coping skills (e.g., grounding, relaxation, communication). Reflecting on these hotline calls, I learned sometimes the most appropriate intervention is to put off intervening and to make space for callers to let out their emotions and to be heard. One major challenge I experienced was maintaining the flow of dialogues, which was more difficult given that the support was provided by texts, which was not instant information exchange. Nevertheless, the convenience it offers cannot be overlooked, especially at a time when phone access might be limited to the frontline health providers.

References

1. Frias L. Wuhan is running low on food, hospitals are overflowing, and foreigners are being evacuated as panic sets in after a week under coronavirus lockdown. Business Insider, January 30,2020 [cited 2020 Apr 28]. https://www.businessinsider.com/no-food-crowded-hospitals-wuhan-first-week-in-coronavirus-quarantine-2020-1

2. BBC News. Wuhan coronavirus lockdown: “We have enough food to last 10 days,” January 24, 2020 [cited 2020 Apr 28]. https://www.bbc.com/news/world-asia-china-51236450

3. Zhong R. $9 cabbages, emergency pork: coronavirus tests in China on food. New York Times, February 4, 2020 [cited 2020 Apr 28]. https://www.nytimes.com/2020/02/04/business/china-coronavirus-food-prices.html

4. Chinese Psychological Society. Guidelines on providing hotline psychosocial support for disease outbreak, January 31,2020 [in Chinese] [cited 2020 Feb 8]. https://mp.weixin.qq.com/s/VIH5Bc0we6qG21neDzdOyQ

5. Chinese Psychological Society. Tiered psychosocial support interventions, March 2, 2020 [in Chinese] [cited 2020 Mar 3]. https://mp.weixin.qq.com/s/SJHE5V2nC1denT7BeolnaQ

6. Chinese Psychological Society. Academic professional association’s psychosocial support response: Chinese Psychosocial Society as an example, April 18, 2020 [in Chinese] [cited 2020 Apr 28]. https://mp.weixin.qq.com/s/mgTQnWgTwPBMYQzgvzvoxw

7. Wang Y. The Chinese suicides prevented by AI from afar. BBC News, November 9, 2019 [cited 2020 Apr 28]. https://www.bbc.com/news/technology-50314819

8. Xia H. Xinhua headlines: mental health services deployed across China in fight against epidemic, February 12, 2020 [cited 2020 Apr 28]. http://www.xinhuanet.com/english/2020-02/12/c_138777329.htm

9. People News. Ministry of Education Huazhong University and Beijing Normal University together establish psychosocial hotline platform, February 24, 2020 [cited 2020 Apr 28]. http://edu.people.com.cn/n1/2020/0224/c367001-31601828.html

10. Ma MY. Hubei Psychological Consultant Association has a 5-year plan to provide psychosocial support to the public, May 1, 2020 [in Chinese] [cited 2020 May 2]. http://hbrbshare.hubeidaily.net/hbshare/news/detail_index.html?contentType=5&contentId=686863&cId=0&tencentShare=1&from=groupmessage&isappinstalled=0

11. Xinhua. Mental health services deployed across China in fight against epidemic, February 13, 2020 [cited 2020 Apr 28]. http://www.chinatoday.com.cn/ctenglish/2018/zdtj/202002/t20200213_800192812.html

12. Liu S, Yang L, Zhang C, Xiang YT, Liu Z, Hu S, et al. Online mental health services in China during the COVID-19 outbreak. Lancet Psychiatry. 2020;7:e17–8. PubMed https://doi.org/10.1016/S2215-0366(20)30077-8

13. Yong Xin Kang Yi Hotline Organization. Overview of Yong Xin Kang Yi hotline organization [in Chinese] [cited 2020 Feb 25]. https://mp.weixin.qq.com/s/uaP64ezYHGgBUYvR8gSiKw
